# γ-Tubulin Complexes and Fibrillar Arrays: Two Conserved High Molecular Forms with Many Cellular Functions

**DOI:** 10.3390/cells10040776

**Published:** 2021-04-01

**Authors:** Jana Chumová, Hana Kourová, Lucie Trögelová, Geoffrey Daniel, Pavla Binarová

**Affiliations:** 1 Institute of Microbiology of the Czech Academy of Sciences, Vídeňská1083, 142 20 Prague, Czech Republic; jana.chumova@biomed.cas.cz (J.C.); kourova@biomed.cas.cz (H.K.); lucie.trogelova@biomed.cas.cz (L.T.); 2 Department of Biomaterials and Technology/Wood Science, Swedish University of Agricultural Sciences, 750-07 Uppsala, Sweden; geoffrey.daniel@slu.se

**Keywords:** microtubules, plants, gamma-tubulin, gamma-tubulin complexes, fibrillar arrays, nucleation, signaling, sequestration

## Abstract

Higher plants represent a large group of eukaryotes where centrosomes are absent. The functions of γ-tubulin small complexes (γ-TuSCs) and γ-tubulin ring complexes (γ-TuRCs) in metazoans and fungi in microtubule nucleation are well established and the majority of components found in the complexes are present in plants. However, plant microtubules are also nucleated in a γ-tubulin-dependent but γ-TuRC-independent manner. There is growing evidence that γ-tubulin is a microtubule nucleator without being complexed in γ-TuRC. Fibrillar arrays of γ-tubulin were demonstrated in plant and animal cells and the ability of γ-tubulin to assemble into linear oligomers/polymers was confirmed in vitro for both native and recombinant γ-tubulin. The functions of γ-tubulin as a template for microtubule nucleation or in promoting spontaneous nucleation is outlined. Higher plants represent an excellent model for studies on the role of γ-tubulin in nucleation due to their acentrosomal nature and high abundancy and conservation of γ-tubulin including its intrinsic ability to assemble filaments. The defining scaffolding or sequestration functions of plant γ-tubulin in microtubule organization or in nuclear processes will help our understanding of its cellular roles in eukaryotes.

## 1. Introduction

Microtubules are dynamic tubular polymers composed of α,β-tubulin heterodimers with diverse functions in cell division, cell transport processes, organelle positioning and many other cellular functions. The spatio–temporal control of microtubule formation is a prerequisite for the assembly of specific microtubular arrays and for the proper functioning of the microtubular cytoskeleton. Microtubules are nucleated and organized from microtubule-organizing centers (MTOCs) such as centrosomes in metazoans or spindle pole bodies in fungi. Centrosome equivalents are still present in ancient land plants like ferns, mosses and liverworts [1], while higher plants represent a large group of eukaryotes that lack centrosomes in all somatic and gametic cells.

## 2. Acentrosomal Nucleation of Microtubules Found in Plants Is a Universal Phenomenon in Eukaryotic Cells

**Figure 1:**
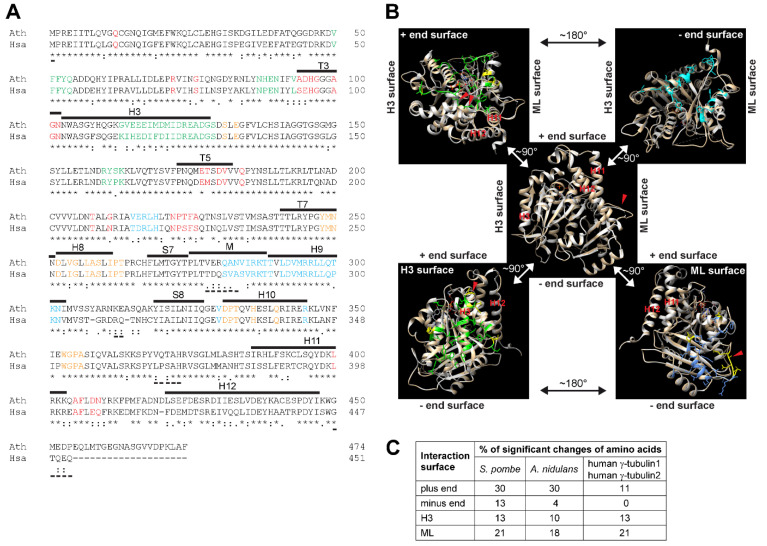
(**A**) The protein sequence alignment of *Arabidopsis* and human γ-tubulins. Amino acids corresponding to those involved in longitudinal and lateral contacts in α- and β-tubulins are colored: red—plus end surface; orange—minus end surface; green—H3 surface; azure—ML surface. Secondary structures or loops are marked with lines above sequences according to human γ-tubulin PDB ID 3cb2a: H—helix; S—β-sheet; T/M—loop. Identical amino acids are marked under sequences according to ClustalW (T-Coffee) with an asterisk, conserved substitutions of the same size and hydropathy with colon, and semi-conserved substitutions of similar size or hydropathy with dot. Underscore under sequences marks amino acids not visible in PDB ID 3cb2a structure: Ath—γ-tubulin1 from *Arabidopsis thaliana*; has—γ-tubulin1 from *Homo sapiens*. (**B**) Comparison of protein structures of *Arabidopsis* and human γ-tubulin1. Cartoon representations of a protein structure model of *Arabidopsis* γ-tubulin1 obtained from a Swiss model (tan) aligned with PDB ID 3cb2a human γ-tubulin1 (white) using Chimera. Amino acids that differed significantly between *Arabidopsis* and human γ-tubulin1 are marked in yellow (semi-and non-conservative T-Coffee). In the center, there is a marked orientation of plus end and minus end surfaces and H3 and ML surfaces needed for longitudinal and lateral interactions, respectively; helices H11 and H12, H9-S8 loop (red arrowhead). Upper left corner—amino acids involved in longitudinal interactions at a plus end surface (green); changed amino acids are generally smaller and/or less polar than those of human γ-tubulin1; change of HWY motif (red arrowheads); upper right corner—amino acids involved in longitudinal interactions at minus end surface (cyan) includes no significantly different amino acids; bottom left corner—amino acids involved in lateral interactions on H3 surface (green); His forming a bulge in helix H3 (red arrowhead) is present in *Arabidopsis* γ-tubulin1, while it is absent in human γ-tubulin; bottom right corner—amino acids involved in lateral interactions on ML surface (cornflower blue); changed amino acids in *Arabidopsis* γ-tubulin1 are larger with only one exception (red arrowhead). GDP (orange stick). (**A**,**B**) Adapted with permission from ref. [3] Copyright 2021 Elsevier. (**C**) Sequence homology at interaction surfaces of γ-tubulin. Significant changes of amino acids (%) in *Homo sapiens* γ-tubulin1/2, *Schizosaccharomyces pombe* and *Aspergillus nidulans* γ-tubulin compared with γ-tubulin1 of *Arabidopsis thaliana*.

γ-Tubulin is a ubiquitous and highly conserved member of the eukaryotic tubulin family [2]. Duplication of the γ-tubulin gene occurred during evolution and γ-tubulin genes *TubG1* and *TubG2* in human and in *Arabidopsis* encode proteins with 98% identity. High conservation at the sequence and structural levels was demonstrated for *Arabidopsis* and human γ-tubulin1 [3]. *Arabidopsis* γ-tubulin1 and human γ-tubulin1 protein sequences share 74 and 86% identity and similarity, respectively (Figure 1A). Amino acids of α-tubulin and β-tubulin, responsible for longitudinal interactions in microtubular protofilaments, are preserved for human γ-tubulin1 and based on the similarity of the residues, an ability of γ-tubulin to form oligomers was suggested [4]. The protein structure of human γ-tubulin was solved by X-ray crystallography [5] and homology modelling was possible due to the high sequential similarity of *Arabidopsis* and human γ-tubulins [3] (Figure 1B). Comparison of the protein sequences of *Arabidopsis* γ-tubulin1 and human γ-tubulin1 showed that the corresponding amino acids required for longitudinal interactions are similar between both γ-tubulins. As compared to conserved surfaces for longitudinal interactions, there are more significant differences between *Arabidopsis* and human γ-tubulin1 concerning the part of the molecule responsible for lateral interactions [3] (Figure 1A,B). Sequences for the longitudinal interactions of γ-tubulin of *S. pombe* and *A. nidulans* showed lower homology with *Arabidopsis* and human γ-tubulins (Figure 1C).

γ-Tubulin is predominantly localized at centrosomes and spindle pole bodies and its specialized role in microtubule nucleation is generally accepted [6,7]. In acentrosomal plant cells, γ-tubulin is present in the cytosol, predominantly associating with microtubular arrays [8]. It is also associated with cytosolic membranes, enriched at the nuclear envelope and a minor part of the γ-tubulin cellular pool is found in nuclei [9,10,11]. Plant γ-tubulin is essential for microtubule nucleation from dispersed γ-tubulin positive sites [12,13].

The acentrosomal nucleation of microtubules was found as a universal phenomenon not only in the absence of centrosomes but also in centrosome-equipped eukaryotic cells. A minor portion of γ-tubulin is present with non-centrosomal sites in animal cells. A subset of microtubules is nucleated from preexisting microtubules, nuclear membrane, Golgi membrane, or kinetochores [14,15,16]. The non-centrosomal microtubule nucleation pathway is important, specifically in large differentiated animal cells like oocytes, epidermal cells and neurons [17,18]. Spindle pole body-independent MTOCs on the nuclear surface nucleate microtubules in fission yeasts [19]. However, compared to well-characterized microtubule organization and dynamics, the microtubule nucleation and early stages of microtubule formation are less understood.

## 3. Complexes of γ-Tubulin with γ-Tubulin Complex Proteins GCPs Are the Best-Established Microtubule Nucleators

The widespread role of γ-tubulin in microtubule nucleation and the mechanisms of microtubule nucleation by γ-tubulin complexes was recently reviewed [6]. There is, however, a growing amount of evidence for MTOC proteins with functions in nucleation, anchoring or regulation. A minimal number of MTOCs components are required for centrosomal and acentrosomal microtubule nucleation in metazoans and fungi [20]. The higher plants represent a large group of eukaryotes where centrosomes are absent. However, the mechanisms of plant acentrosomal microtubule nucleation is still not well understood, and only rarely discussed in the context of metazoans or fungi, apart from a recent review by Lee and Liu [21].

Budding yeasts contain only two GCPs, GCP2 and GCP3, which together with two γ-tubulins form the tetrameric γ-tubulin small complexes—γ-TuSCs. An additional three GCPs, GCP4,5,6 are present in other eukaryotes and together with γ-tubulin compose γ-tubulin ring complexes—γ-TuRCs. γ-TuRCs were described in *Xenopus* and *Drosophila* more than two decades ago [22,23] and a template and protofilament hypothesis of microtubule nucleation from γ-TuRC was suggested [24]. The ability of γ-tubulin monomers to laterally self-associate while in curved conformation was first observed in the X-ray crystal structure of human γ-tubulin [4]. This observation supports the hypothesis of γ-TuRC as a template where γ-tubulin longitudinal interaction with αβ-tubulin stabilizes weak lateral interactions between αβ-tubulins in protofilaments.

MZT1 (Mozart1) and MZT2 (Mozart2, known also as GCP8) proteins, and the WD-repeat protein NEDD1 are other ubiquitous components of γ-TuRC [25]. The γ-tubulin complexes are directed to sites of microtubule nucleation through interactions with attachment and regulatory factors. In animal cells, centrosomin motif 1 CM1-containing protein CDK5RAP2 belongs to the attachment factor of centrosomal scaffold proteins [26]. CM1 proteins Spc110p and MTO1,2 of budding and fission yeasts, respectively, interact with γ-tubulin complexes and have an essential role in the assembly of these complexes into higher-order structures for nucleation [27,28].

Native γ-TuRCs preassembled in the cytoplasm of vertebrates show only low microtubule nucleation efficiency due to their open ring conformation and asymmetrical geometry that do not provide an optimal template for microtubule nucleation [29]. Three independent electron microscopy studies of vertebrate γ-TuRC showed an open and closed conformation of the complexes and suggested a possible regulatory mechanism for microtubule nucleation by γ-TuRC closure [29,30,31]. The models show that two molecules of MZT1 form stable folds with the N-terminal part of GCP6 and one copy of GCP3, and form a lumenal bridge, a belt-like multiprotein scaffold of γ-TuRC. One molecule of actin is an integral component of this structural scaffold. However, the absence of the lumenal bridge did not affect the nucleation efficiency of reconstituted γ-TuRC and a function of the scaffolding in self-assembly of regulatory interfaces was suggested [32]. Single molecule microtubule nucleation assays performed with native complexes purified from *Xenopus* egg extracts confirmed that γ-TuRCs enhance microtubule nucleation by promoting the lateral association of aβ-tubulin molecules and a conformational switch of γ-TuRC was suggested as a result of nucleation [33]. The association of seven tubulin dimers was efficient as a minimal nucleus for microtubule nucleation from γ-TuRCs [30].

There is also a growing number of proteins involved in the regulation of the structure and function of γ-TuRCs. Protein RUVB AAA ATPase functions as a co-chaperone in the assembly of γ-TuRCs in human cells and assists in the reconstitution of the complex from the components co-expressed in heterologous systems [34]. A processive microtubule polymerase XMAP215 associates with γ-TuRCs and promotes the nucleation of microtubules [33].

Plant γ-tubulin interacts with homologues of γ-TuRC members GCP2-6, NEDD1, GIP1,2/MZT1 [3,11,35,36] and from this, a role of γ-tubulin complexed with GCPs in plant acentrosomal microtubule nucleation and organization was suggested [37,38,39]. MZT1 homologues GIP1 and GIP2 localize at sites of microtubule nucleation with the outer nuclear envelope [40]. In human cells, MZT1 binds to the N-terminal extension of GCP3,5 and 6 and targets the γ-tubulin complexes to MTOCs, most probably through interaction with NEDD1 and the CM1 centrosomal scaffold protein CDK5RAP2 [41]. Similarly, MZTs form an efficient microtubule nucleator with CM1 proteins MTO1/2 and small γ-tubulin complex in fission yeasts [27]. Plant cells lack proteins with the CM1 domain due to the absence of centrosomes and GIP1 and GIP2 organize acentrosomal microtubule nucleators most likely through the interaction with plant specific attachment/scaffolding proteins. Furthermore, GIP1 and GIP2 show, in addition to microtubule nucleation, plant specific functions in maintaining centromere architecture and nuclei organization [42].

Although γ-tubulin is considered a universal microtubule nucleator, there are also reports indicating that γ-tubulin is not essential for microtubule nucleation. While the knock-down of γ-tubulin reduces the level of microtubule nucleation from centrosomes and affects microtubular functions, the formation of microtubules is not completely abolished [43,44]. In addition to kinetically dominant γ-tubulin-dependent microtubule nucleation, other nucleation pathways may exist. Perinuclear non-centrosomal MTOCs in *Drosophila* fat body cells assemble microtubules independently of γ-tubulin through the action of nesprin homologue Msp300, patronin and a member of XMAP215 microtubule polymerase family [45]. The centrosome-independent nucleation of microtubules during neuronal axon branching is mediated by the microtubule-associated protein SSNA1 [46]. SSNA1 assembles into fibrils by head-to-tail mechanisms and the fibrillar oligomers attach as a scaffold along single protofilaments guiding them away from the microtubule. Data on the function of SSNA1 in nucleation and branching of microtubules in axons suggest that template-driven nucleation pathways other than γ-TuRC may exist specifically in the large acentrosomal cells.

## 4. Ability of γ-Tubulin to Assemble Fibrillar Arrays Is Conserved in Plants and Animals

**Figure 2:**
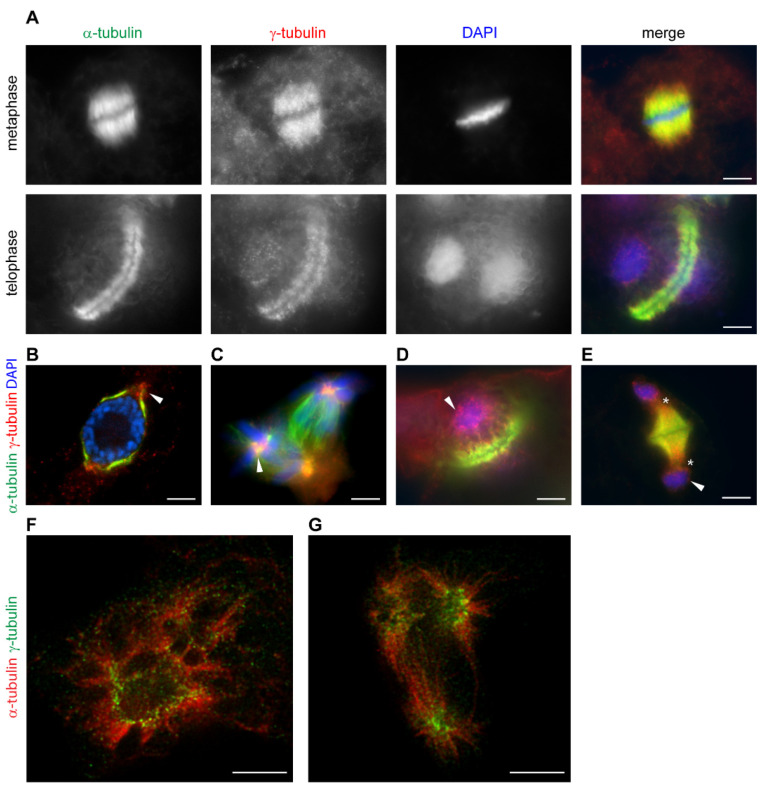
γ-Tubulin localizes with mitotic microtubular arrays and a nuclear envelope in a cell cycle-dependent manner in acentrosomal plant cells. (**A**–**G**): Immunofluorescence labelling of γ-tubulin and α-tubulin in *Arabidopsis* cells—(**A**) γ-Tubulin localizes with microtubules of mitotic spindle and phragmoplast. (**B**–**E**): γ-Tubulin localization in cells treated with roscovitine—(**B**) γ-Tubulin forms condensated protrusions at polar regions in the vicinity of nuclei in cells arrested at G2/M (arrowhead); (**C**) γ-Tubulin foci in centers of chromosomal asters (arrowhead) of a multipolar spindle of *Vicia faba*; (**D**,**E**) γ-Tubulin is localized with minus ends of microtubules of aberrant phragmoplasts, often extending into the cytoplasm (asterisks) and patches of γ-tubulin are observed with newly formed nuclei (arrowhead); (**F**,**G**): STED (stimulated emission depletion) microscopy images of roscovitine- and taxol-treated cells of *Arabidopsis*. (time-gated continuous wave STED, 660 nm depletion laser, deconvolution by Huygens); (**F**) γ-Tubulin fibrillar arrays accumulate with nuclei in cells arrested at G2/M; (**G**) γ-Tubulin fibrillar arrays are enriched in the centers of chromosomal asters of multipolar mitosis. (**A**–**E**): Olympus Cell-R microscopy—(**F**,**G**): super-resolution Leica TCS STED 3X microscope. Scale bars: 5 µm (**A**–**G**).

The ability of γ-tubulin to form dimers and oligo/polymers was suggested based on the preservation of αβ-tubulin surfaces for longitudinal and lateral interactions in human γ-tubulin [5]. γ-Tubulin shows high affinity to αβ-tubulins, γ-tubulin peptides associate with microtubules along the polymer length and the association does not interfere with the microtubule assembly in vitro [47]. γ-Tubulin strongly decorates microtubules in acentrosomal plant cells [8,10] (Figure 2A). The high affinity of γ-tubulin to microtubules polymerized from plant extracts was biochemically and microscopically proven, showing patches of γ-tubulin localized along the entire length of microtubules [10]. γ-Tubulin co-distributes with α- and β-tubulins in fractionation experiments and physically interacts with α- and β-tubulins in the soluble cellular pool of porcine brain and *Arabidopsis* [10,48]. Altogether, these data suggest that the interactions of α- and β-tubulins with γ-tubulin in cells is more complex than expected for the physical contacts during microtubule nucleation from γ-TuRCs.

Interaction of γ-tubulin with α- and β-tubulins, irrespective of the size of γ-tubulin complexes, suggested the presence of other high molecular forms of γ-tubulin apart from γ-TuSC and γ-TuRC [10,48]. Size-heterogeneous large molecular forms of γ-tubulin were demonstrated in fractions of proteins associated with neuronal microtubules [49]. The presence of GCPs with a specific fraction of γ-tubulin complexes suggested that only a subset of the large molecular forms of γ-tubulin is represented by γ-tubulin complexes with GCPs [3]. The ability of γ-tubulin to oligomerize was demonstrated by non-denaturing PAGE in neuronal cell extracts, and oligomerization was proven for γ-tubulin immunopurified from a specific fraction of proteins associated with brain microtubules [3,48].

The ability of γ-tubulin to assemble oligomers/polymers, as biochemically shown and by structure-based predictions, was also confirmed microscopically. The recombinant human γ-tubulin expressed in *Escherichia coli* is prone to aggregation, but when purified γ-tubulin was incubated with the CCT chaperone and ATP, fibrillar arrays were observed in addition to aggregates [50] (Table 1). The proper assembly and function of eukaryotic tubulins requires chaperone mediated-folding and chaperones, and CCT were copurified with αβ-tubulin and γ-tubulin [51,52]. While endogenous γ-tubulin is present in animal and plant extracts in the form of dimers [3,48,53], γ-tubulin expressed in the baculovirus system is monomeric [4,53,54]. Although monomers may not represent the natural form of the γ-tubulin, their binding to microtubules in vitro in a salt-dependent and microtubule end-dependent manner is preserved [53]. γ-Tubulin coexpressed with GCPs in baculovirus reconstitutes γ-TuRCs that are capable of microtubule nucleation [32].

The fibrillar arrays of γ-tubulin were also demonstrated in human U2OS cells and fibrils of γ-tubulin assembled from bacterially expressed human γ-tubulin [55] (Table 1). Biochemical characterization of native endogenous γ-tubulin purified from *Arabidopsis*, human U2OS and porcine brain cell extracts showed dimers and oligo-polymers of γ-tubulin [3]. TEM analyses of the purified γ-tubulin showed short filaments mostly with a double parallel protofilament substructure that either aggregated or assembled into longer bundled fibrillar arrays (Table 1). The fibrillar arrays showed an almost parallel alignment, although they were occasionally helically intertwined. AFM (atomic force microscopy) analyses of the γ-tubulin fibrillar assemblies revealed the most frequent width of filaments as 8.5 nm which was consistent with the width estimated from TEM analyses (~6 × 9 nm in a cross section) [3]. The diameter of human γ-tubulin filament reported by Rossello [55] and Pouchucq [50] is also comparable (Table 1). The assembly of filaments demonstrated in vitro for native γ-tubulin purified from *Arabidopsis*, human cells and porcine brain was concentration dependent but not GTP dependent. Compared to GTP-hydrolyzing α-tubulin or prokaryotic FtsZ tubulin, the acidic residues in the catalytic sites of plant and animal γ-tubulin are absent [3,5]. Similarly, GTP was not required for the polymerization of purified human γ-tubulin in the experiments conducted by Pouchucq [50] and Rossello [55].

## 5. γ-Tubulin Is a Microtubule Nucleator in the Absence of Other γ-TuRC Components

*Arabidopsis* mutants without a functional GCP6 homologue showed impaired assembly of γ-TuRC [57]. This is consistent with the fact that GCP6 carries an insertion domain, which acts as a scaffold for GCP2 and GCP5 proteins in γ-TuRC assemblies [29]. Analyses of *Arabidopsis* GCP6 mutants showed that γ-TuRCs are indispensable for spindle pole organization. In contrast, the nucleation of microtubules of the cytokinesis specific apparatus phragmoplast takes place in a γ-TuRC-independent manner [57]. However, it is known that γ-tubulin is essential for the nucleation of phragmoplast microtubules [12,13]. γ-Tubulin also associates with microtubules in cells with an impaired function of γ-TuRCs and nucleation of specific microtubular arrays of plants may be promoted by γ-tubulin which is not complexed with GCPs [57]. Large molecular forms of γ-tubulin from *Arabidopsis* extracts reduce the critical level of αβ-tubulin dimers required for microtubule nucleation [10]. STED microscopy showed that fibrillar γ-tubulin structures localize with microtubular arrays of the mitotic spindle and phragmoplast and with the nuclear envelope; e.g., with dispersed sites of acentrosomal plant microtubule formation in *Arabidopsis* cells [3].

Compared with animals, γ-tubulin is more abundant in plant cells, presumably due to their acentrosomal nature [3,10]. While γ-tubulin protein levels are similar with those of α,β-tubulins or actin, the GCPs components of γ-TuRC belong to the least abundant proteins in the cells [58]. In addition to the established role for γ-tubulin in stabilizing αβ-tubulin lateral bonds via the γ-TuRC template, other types of interaction of γ-tubulin with αβ-tubulin/microtubules are also expected during microtubule formation. Models of spontaneous microtubule nucleation include several rate-limiting steps. In the early phase of microtubule nucleation, αβ-tubulin dimers associate into short double protofilaments, oligomers and nucleation intermediates that seed further the formation of sheets [59]. αβ-Tubulin dimers form short protofilament intermediates independent of their GTP/GDP nucleotide state [60]. Models of the concentration-dependent kinetics of microtubule assembly indicate that the 2–3 laterally associated tubulin binding sites may reduce the kinetic barrier during spontaneous microtubule nucleation in the absence of a specific nucleus [61]. It is tempting to speculate that the γ-tubulin pool of unpolymerized monomers/dimers or the short γ-tubulin protofilaments may assist in the early stages of microtubule nucleation by reducing the kinetic barrier and by promoting the longitudinal or lateral tubulin/tubulin interaction of nucleation intermediates. γ-Tubulin monomers were shown to act as seeds for αβ-tubulin protofilament nucleation with a strong interaction of γ-tubulin with β-tubulin demonstrated [54]. In addition to the involvement of γ-tubulin at the early stages of microtubule formation, γ-tubulin also acts in later stages by being present at the plus ends of microtubules presumably functioning in closure of the seam of nascent microtubules [62].

Microtubule-associated proteins (MAPs) can have an impact on microtubule nucleation by promoting the interaction of tubulins in nucleation intermediates [63]. TPX2 protein recognizes and interacts specifically with αβ-tubulin of oligomer intermediates in microtubule nucleation [64]. Residues responsible for TPX2 interaction with the protofilaments of microtubules are conserved in animal and *Arabidopsis* cells [65,66]. Aurora 1 kinase and its activator TPX2 are co-localized with γ-tubulin on spindle microtubules of *Arabidopsis* forming a gradient from the mid-zone to the growing ends of microtubules at the spindle poles [66]. The contribution of γ-tubulin with TPX2 or with other MAPs in stabilizing nascent nuclei at the growing ends of microtubules may represent an important mechanism in promoting microtubule nucleation.

In order to gain better insight into the cooperation of MAPs with γ-tubulin templates, the effect of protein XMAP215 together with γ-tubulin on microtubule nucleation was studied using life scattering assays [56]. The linear fibrils formed from high concentrations of purified expressed human γ-tubulin promoted nucleation of microtubules. The X-ray crystal structure of human γ-tubulin revealed that monomeric γ-tubulin has a unique ability to form stable lateral interactions with assemblies of γ-tubulin presumably providing a template that can stabilize the lateral interaction between adjacent α,β-tubulins nucleated from γ-TuRC [4]. The docking of the human γ-tubulin crystal structures in 3D helical reconstructions of TEM images of the fibrillar arrays observed by King et al. [56] suggested that the fibrils are likely formed by γ-tubulins lateral alignment on their long axis. However, how microtubules are nucleated from the fibrillar arrays of γ-tubulin with five-fold symmetry is not fully understood. Microtubule polymerase XMAP215 functions additively with laterally associated arrays of γ-tubulin in promoting microtubule nucleation [56]. γ-Tubulin filaments were also observed in in vitro reconstitution experiments with purified recombinant human γ-tubulin by Tawani et al. [33] (Table 1). The linear polymers are thought to be formed by the lateral interaction of γ-tubulin molecules and promote microtubule nucleation by providing a binding surface for αβ-tubulin polymerization.

## 6. An Ability of γ-Tubulin to Form Filaments May Be Important for Other Less Defined Cellular Functions

In addition to the most intensively studied role of γ-tubulin in microtubule nucleation, γ-tubulin also functions in cell cycle regulation and nuclear processes across eukaryotes (recently reviewed by Oakley [67], Corvaisier and Kristensson [68], and Chumova [69]). Interaction between fibrillar γ-tubulin and lamin B, as well as a function of the γ-tubulin fibrillar network in the organization of nuclei were suggested [55]. Interaction between γ-tubulin and the LINC complex component SUN protein of inner nuclear envelope was indicated in plants [69] and the interaction between γ-tubulin and transcription factors E2Fs and its function in the regulation of the expression of cell cycle genes was shown in animal and plant cells [70,71].

The localization of plant γ-tubulin at dispersed sites is regulated by cell cycle signaling [72]. γ-Tubulin is present with microtubular mitotic and cytokinetic arrays and accumulates in the vicinity of the nuclear envelope in a cell cycle-dependent manner (Figure 2A). During the breakdown of the cell cycle, γ-tubulin is dramatically relocated. In cells treated with roscovitine, an inhibitor of cyclin-dependent kinases, patches of condensed γ-tubulin accumulated in polar-regions in close vicinity of the persistent nuclear envelope of cells arrested at the G2/M interface (Figure 2B). γ-Tubulin diminished under roscovitine treatment from the mitotic microtubular spindle and accumulated in foci in centers of chromosomal asters in multipolar mitosis (Figure 2C). In cells with aberrant phragmoplast, γ-tubulin is enriched with minus ends of microtubules and is present with the reformed nuclear envelope (Figure 2D,E). STED microscopy showed that the patches of γ-tubulin accumulated in roscovitine-treated cells with persistent nuclear envelope and in centers of multipolar chromosomal asters were composed of fibrillar γ-tubulin structures [3] (Figure 2F,G). γ-Tubulin was also shown to interact with stress signaling MAP kinases in *Arabidopsis*. MAP kinase MPK6 interacts with γ-tubulin but neither γ-tubulin nor GCPs were phosphorylated by the kinase and a scaffolding role of γ-tubulin in plant MAP kinases signaling was suggested [73].

In addition to the fine γ-tubulin filaments shown by super-resolution microscopy, more robust γ-tubulin rod-like structures are sporadically found in nuclei, the perinuclear area and the cytoplasm of non-dividing cells of *Arabidopsis* [3]. Similar robust fibrillar structures of γ-tubulin were observed in non-dividing mammalian cells [74]. Super-resolution microscopy analyses indicated that the formation of the rods may reflect the tendency of γ-tubulin filaments to aggregate in vitro [3,50]. γ-Tubulin is detected together with other fibrillar proteins as a ubiquitous component of aggresomes in neurodegenerative diseases [75]. γ-Tubulin associated with brain microtubules forms oligomers [48] and aggregating fibrillar arrays of γ-tubulin may be utilized in the generation of aggresomes and inclusion bodies in the brain. Unfolded recombinant γ-tubulin microinjected into single-cell zebrafish embryos forms large intracellular aggregates resembling perinuclear aggresomes [50]. Most proteins recognized by CCT chaperones show topologies prone to aggregation usually through the recognition of β-strand regions [76]. γ-Tubulin in acentrosomal higher plants has a specific C-terminal extension with β-strands indicated in the I-TASSER model [3].

The condensation of intrinsically disordered proteins via liquid–liquid phase separation (LLS) enhances the rates and efficiency of compartmentalized cellular processes. Separated condensates promote the assembly of cytoskeletal proteins through the formation of a concentrated phase; TPX2 and tubulin form co-condensates that promote the assembly of microtubules in both the cytoplasm and in vitro [77]. TPX2 co-localized with γ-tubulin in *Arabidopsis* cells on microtubules and in perinuclear/nuclear areas [78] and we can only speculate that TPX2 may recruit γ-tubulin together with α,β-tubulin as a condensate to attain the higher concentrations required for γ-tubulin filament formation and microtubule nucleation. A concept of phase separation in promoting acentrosomal spindle formation has recently emerged. Spherical protrusions at acentrosomal spindle poles of mammalian oocytes formed by condensation locally sequester and mobilize factors regulating spindle microtubule formation within the cytoplasm [79]. During plant acentrosomal spindle organization, γ-tubulin forms patches in the vicinity of the nuclear envelope (Figure 2B) or with the spindle [10], often protruding into the cytoplasm at the spindle pole area (Figure 1B). Whether γ-tubulin by itself or with other proteins use mechanisms of phase separation to concentrate or sequester the factors required for microtubule nucleation and the organization of microtubular arrays from dispersed MTOCs remains to be elucidated. γ-Tubulin is present in the nuclei and its function with E2F transcription factors was shown in both the animal and plant cells [70,71]. γ-Tubulin was also found in DNA repair foci [80]. The DNA repair protein Rad52 assembles in liquid droplets to concentrate tubulin to promote the formation of intranuclear microtubule filaments that move damaged DNA to the nuclear periphery for repair [81].

## 7. Concluding Remarks

*Arabidopsis* and human γ-tubulin are conserved on the sequence and structural levels. A higher abundance of γ-tubulin in acentrosomal plant cells enables the purification of native γ-tubulin and to prove its intrinsic ability to polymerize filaments and demonstrate the fibrillar arrays of γ-tubulin in *Arabidopsis* cells. The plant homologues of GCPs physically interact with γ-tubulin and a conserved role of γ-TuRC in microtubule nucleation is suggested [21,69]. The nucleation of plant microtubules, at least in some microtubular arrays, may take part in a γ-tubulin-dependent manner, but does not require functional γ-TuRCs [57]. In this review, we provide an overview of the higher molecular forms of γ-tubulin and their functions in plants, metazoans and fungi. Dual roles for γ-tubulin are suggested: (i) γ-tubulin forms a template through the lateral interaction of monomers in γ-TuRCs and perhaps in linear fibrillar arrays and provides a platform for interaction with α,β-tubulins and for the stabilization of their lateral interaction in microtubule nucleation. γ-Tubulin is also a microtubule nucleator without forming complexes with GCPs and may promote the early and late stages of microtubule formation by itself or in collaboration with MAPs. However, the molecular mechanisms behind the process remains to be elucidated; (ii) γ-Tubulin with an ability to assemble dimers and short protofilaments in both plant- and animal cells belongs to the filament forming tubulins. Scaffolding and sequestration functions that are well established for prokaryotic filament forming tubulins may be behind numerous interactions of γ-tubulin and its functions not only with microtubules but also in nuclear and other cellular processes. γ-Tubulin preserves the properties of both eukaryotic and prokaryotic tubulins. Comparative studies of the clade of eukaryotic tubulin showed the highest similarity of γ-tubulin to β-tubulins and BtubA and BtubB of *Prosthecobacter dejongeii* [3]. The horizontal transfer of BtubA and BtubB genes early on after the initial duplication of homologue pairs was suggested and BtubA/B form filaments and tubular structures with a five-fold symmetry [82].

There are still more questions than answers concerning γ-tubulin cellular functions in microtubule nucleation, in nuclear processes, and its possible participation in phase separation processes of acentrosomal spindle formation and in aggresome formation. Addressing these questions requires an integrative approach to bring together knowledge from acentrosomal plant cells, animals, and fungi.

## Figures and Tables

**Table 1:** γ-Tubulin fibrillar arrays.

Organism	Endogenous/Recombinant γ-Tubulin	Purification	Polymerization In Vitro or In Situ Analyses	Fibrillar γ-Tubulin Arrays. Microscopy	Biochemical Analyses	Reference
**Human** **γ-tubulin**	His6-human γ-tubulin expressed in *E. coli*	Ni^2+^ affinity resin 20 mM Tris pH 7.9 500 mM NaCl 0.25 μM GTP	In vitro 40 mM K-Hepes 150 mM NaCl no GTP	TEM, filaments (γ-strings)		Rossello et al., 2016 [55]
	Endogenous		In fixed U2OS cells	CLSM, SR-SIM, TEM, immunogold labelling for TEM, filaments (γ-strings) (diameter 4–6 nm)		
	sh-resistant GFP-γ-tubulin		In vivo in U2OS cells	Fluorescence microscopy, filaments (γ-strings)		
**Human** **γ-tubulin1**	Human γ-tubulin expressed in *E. coli*	Ion exchange chromatography column, KCl gradient elution	In vitro 50 mM Tris, pH 7.2 150 mM NaCl no GTP 1 µM γ-tubulin	TEM, amorphous aggregates, fibers (~6.7 nm) (formation promoted by CCT chaperonin +ATP)	Light scattering of 1 µM γ-tubulin in the presence of CCT	Pouchucq et al., 2018 [50]
***Arabidopsis*** ***thaliana*** **γ-tubulin**	Endogenous *Arabidopsis* γ-tubulin	*Arabidopsis* cell extracts in 50 mM·Hepes pH 7.5 75 mM NaCl Imumo purification IP Ath specific anti-γ-tubulin antibody, peptide elution (a)	In vitro 50–100 mM Tris pH 7.5–8.0 no GTP >8 µM γ-tubulin	TEM, short filaments (~6 × 9 nm in cross section), AFM ~8.5 nm width Bundles of filaments, aggregates of short filaments Fluorescence microscopy	Sucrose gradient centrifugation Size exclusion chromatography Native PAGE, Western Blotting	Chumova et al., 2018 [3]
	*Arabidopsis* TubG1-GFP expressed in *Arabidopsis*	IP anti-GFP, low pH glycine elution (b)				
	Endogenous γ-tubulin		In *Arabidopsis* cells, IF of endogenous γ-tubulin for STED	CLSM, pulse-STED		
**Human** **γ-tubulin**	Human TubG1-RFP in U2OS cells	U2OS cell extracts 50 mM·Hepes pH 7.5 75 mM NaCl IP anti-RFP, low pH glycine elution (c)	In vitro 100 mM Tris pH 7.5–8.0 no GTP	TEM, filaments, double protofilament, filament bundles Fluorescence microscopy		
**Porcine** **γ-tubulin**	Endogenous porcine γ-tubulin	Proteins associated with brain microtubules 80 mM Pipes pH 6.8 IP anti-γ-tubulin antibody and peptide elution	No GTP	Oligomers	Native PAGE, WB	
**Human** **γ-tubulin**	Human γ-tubulin TEV-Strep II-6xHis tags, expressed in Sf9 cells (Bac-to-Bac system)	HisTrap HP, gel filtration, 50 mM K-MES pH 6.6 500 mM KCl 1 µM GTP	In vitro 50 mM K-MES pH 6.6 100 mM KCl no GTP 1–2 µM γ-tubulin	TEM, filaments of variable widths, 3D helical reconstruction and docking of human γ-tubulin crystal structure [19], (d)	Size exclusion chromatography	Thawani et al., 2020 [33]
**Human** **γ-tubulin**	γ-tubulin myc-His6 tag, expressed in Sf9 cells (Bac-to-Bac system)	Ni-NTA Superflow resin, gel filtration 50 mM K-MES pH 6.6 500 mM KCl 1 µM GTP	In vitro ≤100 mM KCl with or without GTP >250 nM γ-tubulin	TEM, fibrillar arrays, 3D helical reconstruction, docking of human γ-tubulin crystal structure [19], (e)	No light scattering of 1 µM γ-tubulin	King et al., 2020 [56]

(a) Alternatively, La3+-PEG precipitation was used to enrich high molecular fraction of endogenous *Arabidopsis* γ-tubulin in the input for immunopurification; (b) alternatively, high stringency conditions 0.3% SDS were applied to dissociate proteins associated with *Arabidopsis* γ-tubulin; (c) alternatively, high stringency conditions 0.08% SDS were applied to dissociate proteins associated with human γ-tubulin; (d) filaments of laterally associated γ-tubulin (repeat distance ~54 Å, 4 linear arrays); (e) arrays of laterally associated γ-tubulins, five-fold symmetry (repeat distance ~52 Å).

## Abbreviations

AFM	atomic force microscopy
CLSM	confocal laser scanning microscopy
CM1	centrosomin motif 1
GCP	γ-tubulin complex protein
GIP	GCP3-interacting protein
γ-TuSC	γ-tubulin small complex
γ-TuRC	γ-tubulin ring complex
MAP	microtubule-associated protein
MTOC	microtubule-organizing center
MZT1, 2	Mozart1, 2
SR-SIM	super-resolution structured illumination microscopy
STED	stimulated emission depletion
TEM	transmission electron microscopy

## References

[1] Brown R.C., Lemmon B.E., Horio T. (2004). Gamma-tubulin localization changes from discrete polar organizers to anastral spindles and phragmoplasts in mitosis of *Marchantia polymorpha* L.. Protoplasma.

[2] Stearns T., Evans L., Kirschner M. (1991). Gamma-tubulin is a highly conserved component of the centrosome. Cell.

[3] Chumova J., Trogelova L., Kourova H., Volc J., Sulimenko V., Halada P., Kucera O., Benada O., Kucharova A., Klebanovych A. (2018). gamma-Tubulin has a conserved intrinsic property of self-polymerization into double stranded filaments and fibrillar networks. Biochim. Biophys. Acta Mol. Cell Res..

[4] Inclan Y.F., Nogales E. (2001). Structural models for the self-assembly and microtubule interactions of gamma-, delta- and epsilon-tubulin. J. Cell Sci..

[5] Aldaz H., Rice L.M., Stearns T., Agard D.A. (2005). Insights into microtubule nucleation from the crystal structure of human gamma-tubulin. Nature.

[6] Liu P., Wurtz M., Zupa E., Pfeffer S., Schiebel E. (2021). Microtubule nucleation: The waltz between gamma-tubulin ring complex and associated proteins. Curr. Opin Cell Biol..

[7] Oakley B.R., Oakley C.E., Yoon Y., Jung M.K. (1990). Gamma-tubulin is a component of the spindle pole body that is essential for microtubule function in Aspergillus nidulans. Cell.

[8] Liu B., Marc J., Joshi H.C., Palevitz B.A. (1993). A gamma-tubulin-related protein associated with the microtubule arrays of higher plants in a cell cycle-dependent manner. J. Cell Sci..

[9] Binarova P., Cenklova V., Hause B., Kubatova E., Lysak M., Dolezel J., Bogre L., Draber P. (2000). Nuclear gamma-tubulin during acentriolar plant mitosis. Plant Cell.

[10] Drykova D., Cenklova V., Sulimenko V., Volc J., Draber P., Binarova P. (2003). Plant gamma-tubulin interacts with alphabeta-tubulin dimers and forms membrane-associated complexes. Plant Cell.

[11] Seltzer V., Janski N., Canaday J., Herzog E., Erhardt M., Evrard J.L., Schmit A.C. (2007). Arabidopsis GCP2 and GCP3 are part of a soluble gamma-tubulin complex and have nuclear envelope targeting domains. Plant J..

[12] Binarova P., Cenklova V., Prochazkova J., Doskocilova A., Volc J., Vrlik M., Bogre L. (2006). Gamma-tubulin is essential for acentrosomal microtubule nucleation and coordination of late mitotic events in Arabidopsis. Plant Cell.

[13] Pastuglia M., Azimzadeh J., Goussot M., Camilleri C., Belcram K., Evrard J.L., Schmit A.C., Guerche P., Bouchez D. (2006). Gamma-tubulin is essential for microtubule organization and development in Arabidopsis. Plant Cell.

[14] Espigat-Georger A., Dyachuk V., Chemin C., Emorine L., Merdes A. (2016). Nuclear alignment in myotubes requiRes. centrosome proteins recruited by nesprin-1. J. Cell Sci..

[15] Mishra R.K., Chakraborty P., Arnaoutov A., Fontoura B.M., Dasso M. (2010). The Nup107-160 complex and gamma-TuRC regulate microtubule polymerization at kinetochores. Nat. Cell Biol..

[16] Rivero S., Cardenas J., Bornens M., Rios R.M. (2009). Microtubule nucleation at the cis-side of the Golgi apparatus requiRes. AKAP450 and GM130. Embo. J..

[17] Goshima G., Kimura A. (2010). New look inside the spindle: Microtubule-dependent microtubule generation within the spindle. Curr. Opin Cell Biol..

[18] Sanchez A.D., Feldman J.L. (2017). Microtubule-organizing centers: From the centrosome to non-centrosomal sites. Curr. Opin Cell Biol..

[19] Tran P.T., Marsh L., Doye V., Inoue S., Chang F. (2001). A mechanism for nuclear positioning in fission yeast based on microtubule pushing. J. Cell Biol..

[20] Paz J., Luders J. (2018). Microtubule-Organizing Centers: Towards a Minimal Parts List. Trends Cell Biol..

[21] Lee Y.-R.J., Liu B. (2019). Microtubule nucleation for the assembly of acentrosomal microtubule arrays in plant cells. New Phytol..

[22] Oegema K., Wiese C., Martin O.C., Milligan R.A., Iwamatsu A., Mitchison T.J., Zheng Y.X. (1999). Characterization of two related Drosophila gamma-tubulin complexes that differ in their ability to nucleate microtubdes. J. Cell Biol..

[23] Zheng Y., Wong M.L., Alberts B., Mitchison T. (1995). Nucleation of microtubule assembly by a gamma-tubulin-containing ring complex. Nature.

[24] Erickson H.P., Stoffler D. (1996). Protofilaments and rings, two conformations of the tubulin family conserved from bacterial FtsZ to alpha/beta and gamma tubulin. J. Cell Biol..

[25] Teixido-Travesa N., Villen J., Lacasa C., Bertran M.T., Archinti M., Gygi S.P., Caelles C., Roig J., Luders J. (2010). The gammaTuRC revisited: A comparative analysis of interphase and mitotic human gammaTuRC redefines the set of core components and identifies the novel subunit GCP8. Mol. Biol. Cell.

[26] Zhang J., Megraw T.L. (2007). Proper recruitment of gamma-tubulin and D-TACC/Msps to embryonic Drosophila centrosomes requiRes. Centrosomin Motif 1. Mol. Biol. Cell.

[27] Leong S.L., Lynch E.M., Zou J., Tay Y.D., Borek W.E., Tuijtel M.W., Rappsilber J., Sawin K.E. (2019). Reconstitution of Microtubule Nucleation In Vitro Reveals Novel Roles for Mzt1. Curr. Biol..

[28] Lin T.C., Neuner A., Schlosser Y.T., Scharf A.N., Weber L., Schiebel E. (2014). Cell-cycle dependent phosphorylation of yeast pericentrin regulates gamma-TuSC-mediated microtubule nucleation. eLife.

[29] Liu P., Zupa E., Neuner A., Bohler A., Loerke J., Flemming D., Ruppert T., Rudack T., Peter C., Spahn C. (2020). Insights into the assembly and activation of the microtubule nucleator gamma-TuRC. Nature.

[30] Consolati T., Locke J., Roostalu J., Chen Z.A., Gannon J., Asthana J., Lim W.M., Martino F., Cvetkovic M.A., Rappsilber J. (2020). Microtubule Nucleation Properties of Single Human gammaTuRCs Explained by Their Cryo-EM Structure. Dev. Cell.

[31] Wieczorek M., Urnavicius L., Ti S.C., Molloy K.R., Chait B.T., Kapoor T.M. (2020). Asymmetric Molecular Architecture of the Human gamma-Tubulin Ring Complex. Cell.

[32] Wieczorek M., Ti S.C., Urnavicius L., Molloy K.R., Aher A., Chait B.T., Kapoor T.M. (2021). Biochemical reconstitutions reveal principles of human gamma-TuRC assembly and function. J. Cell Biol..

[33] Thawani A., Rale M.J., Coudray N., Bhabha G., Stone H.A., Shaevitz J.W., Petry S. (2020). The transition state and regulation of gamma-TuRC-mediated microtubule nucleation revealed by single molecule microscopy. eLife.

[34] Zimmermann F., Serna M., Ezquerra A., Fernandez-Leiro R., Llorca O., Luders J. (2020). Assembly of the asymmetric human gamma-tubulin ring complex by RUVBL1-RUVBL2 AAA ATPase. Sci. Adv..

[35] Nakamura M., Ehrhardt D.W., Hashimoto T. (2010). Microtubule and katanin-dependent dynamics of microtubule nucleation complexes in the acentrosomal Arabidopsis cortical array. Nat. Cell Biol..

[36] Nakamura M., Yagi N., Kato T., Fujita S., Kawashima N., Ehrhardt D.W., Hashimoto T. (2012). Arabidopsis GCP3-interacting protein 1/MOZART 1 is an integral component of the gamma-tubulin-containing microtubule nucleating complex. Plant J..

[37] Kong Z., Hotta T., Lee Y.-R.J., Horio T., Liu B. (2010). The gamma-Tubulin Complex Protein GCP4 Is Required for Organizing Functional Microtubule Arrays in Arabidopsis thaliana. Plant Cell.

[38] Nakamura M., Hashimoto T. (2009). A mutation in the Arabidopsis gamma-tubulin-containing complex causes helical growth and abnormal microtubule branching. J. Cell Sci..

[39] Zeng C.J., Lee Y.R., Liu B. (2009). The WD40 repeat protein NEDD1 functions in microtubule organization during cell division in Arabidopsis thaliana. Plant Cell.

[40] Janski N., Masoud K., Batzenschlager M., Herzog E., Evrard J.L., Houlne G., Bourge M., Chaboute M.E., Schmit A.C. (2012). The GCP3-interacting proteins GIP1 and GIP2 are required for gamma-tubulin complex protein localization, spindle integrity, and chromosomal stability. Plant Cell.

[41] Cota R.R., Teixido-Travesa N., Ezquerra A., Eibes S., Lacasa C., Roig J., Luders J. (2017). MZT1 regulates microtubule nucleation by linking gammaTuRC assembly to adapter-mediated targeting and activation. J. Cell Sci..

[42] Batzenschlager M., Lermontova I., Schubert V., Fuchs J., Berr A., Koini M.A., Houlne G., Herzog E., Rutten T., Alioua A. (2015). Arabidopsis MZT1 homologs GIP1 and GIP2 are essential for centromere architecture. Proc. Natl. Acad. Sci. USA.

[43] Hannak E., Oegema K., Kirkham M., Gonczy P., Habermann B., Hyman A.A. (2002). The kinetically dominant assembly pathway for centrosomal asters in Caenorhabditis elegans is gamma-tubulin dependent. J. Cell Biol..

[44] Rogers G.C., Rusan N.M., Peifer M., Rogers S.L. (2008). A multicomponent assembly pathway contributes to the formation of acentrosomal microtubule arrays in interphase Drosophila cells. Mol. Biol. Cell.

[45] Zheng Y., Buchwalter R.A., Zheng C., Wight E.M., Chen J.V., Megraw T.L. (2020). A perinuclear microtubule-organizing centre controls nuclear positioning and basement membrane secretion. Nat. Cell Biol..

[46] Basnet N., Nedozralova H., Crevenna A.H., Bodakuntla S., Schlichthaerle T., Taschner M., Cardone G., Janke C., Jungmann R., Magiera M.M. (2018). Direct induction of microtubule branching by microtubule nucleation factor SSNA1. Nat. Cell Biol..

[47] Llanos R., Chevrier V., Ronjat M., Meurer-Grob P., Martinez P., Frank R., Bornens M., Wade R.H., Wehland J., Job D. (1999). Tubulin binding sites on gamma-tubulin: Identification and molecular characterization. Biochemistry.

[48] Sulimenko V., Sulimenko T., Poznanovic S., Nechiporuk-Zloy V., Bohm K.J., Macurek L., Unger E., Draber P. (2002). Association of brain gamma-tubulins with alpha beta-tubulin dimers. Biochem. J..

[49] Detraves C., Mazarguil H., LajoieMazenc I., Julian M., RaynaudMessina B., Wright M. (1997). Protein complexes containing gamma-tubulin are present in mammalian brain microtubule protein preparations. Cell Motil. Cytoskelet..

[50] Pouchucq L., Lobos-Ruiz P., Araya G., Valpuesta J.M., Monasterio O. (2018). The chaperonin CCT promotes the formation of fibrillar aggregates of gamma-tubulin. Biochim. Biophys. Acta Proteins Proteom..

[51] Moudjou M., Bordes N., Paintrand M., Bornens M. (1996). gamma-Tubulin in mammalian cells: The centrosomal and the cytosolic forms. J. Cell Sci..

[52] Melki R., Vainberg I.E., Chow R.L., Cowan N.J. (1993). Chaperonin-mediated folding of vertebrate actin-related protein and gamma-tubulin. J. Cell Biol..

[53] Vassilev A., Kimble M., Silflow C.D., LaVoie M., Kuriyama R. (1995). Identification of intrinsic dimer and overexpressed monomeric forms of gamma-tubulin in Sf9 cells infected with baculovirus containing the Chlamydomonas gamma-tubulin sequence. J. Cell Sci..

[54] Leguy R., Melki R., Pantaloni D., Carlier M.F. (2000). Monomeric gamma-tubulin nucleates microtubules. J. Biol. Chem..

[55] Rossello C.A., Lindstrom L., Glindre J., Eklund G., Alvarado-Kristensson M. (2016). Gamma-tubulin coordinates nuclear envelope assembly around chromatin. Heliyon.

[56] King B.R., Moritz M., Kim H., Agard D.A., Asbury C.L., Davis T.N. (2020). XMAP215 and gamma-tubulin additively promote microtubule nucleation in purified solutions. Mol. Biol. Cell.

[57] Miao H., Guo R., Chen J., Wang Q., Lee Y.J., Liu B. (2019). The gamma-tubulin complex protein GCP6 is crucial for spindle morphogenesis but not essential for microtubule reorganization in Arabidopsis. Proc. Natl. Acad. Sci. USA.

[58] Beck M., Schmidt A., Malmstroem J., Claassen M., Ori A., Szymborska A., Herzog F., Rinner O., Ellenberg J., Aebersold R. (2011). The quantitative proteome of a human cell line. Mol. Syst. Biol..

[59] Voter W.A., Erickson H.P. (1984). The kinetics of microtubule assembly. Evidence for a two-stage nucleation mechanism. J. Biol. Chem..

[60] Mozziconacci J., Sandblad L., Wachsmuth M., Brunner D., Karsenti E. (2008). Tubulin dimers oligomerize before their incorporation into microtubules. PLoS ONE.

[61] Rice L.M., Moritz M., Agard D.A. (2020). Microtubules form by progressively faster tubulin accretion, not by nucleation-elongation. bioRxiv.

[62] Raynaud-Messina B., Merdes A. (2007). gamma-tubulin complexes and microtubule organization. Curr. Opin. Cell Biol..

[63] Roostalu J., Surrey T. (2017). Microtubule nucleation: Beyond the template. Nat. Rev. Mol. Cell Biol..

[64] Roostalu J., Cade N.I., Surrey T. (2015). Complementary activities of TPX2 and chTOG constitute an efficient importin-regulated microtubule nucleation module. Nat. Cell Biol..

[65] Gruss O.J., Wittmann M., Yokoyama H., Pepperkok R., Kufer T., Sillje H., Karsenti E., Mattaj I.W., Vernos I. (2002). Chromosome-induced microtubule assembly mediated by TPX2 is required for spindle formation in HeLa cells. Nat. Cell Biol..

[66] Petrovska B., Cenklova V., Pochylova Z., Kourova H., Doskocilova A., Plihal O., Binarova L., Binarova P. (2012). Plant Aurora kinases play a role in maintenance of primary meristems and control of endoreduplication. New Phytol..

[67] Oakley B.R., Paolillo V., Zheng Y. (2015). gamma-Tubulin complexes in microtubule nucleation and beyond. Mol. Biol. Cell.

[68] Corvaisier M., Alvarado-Kristensson M. (2020). Non-Canonical Functions of the Gamma-Tubulin Meshwork in the Regulation of the Nuclear Architecture. Cancers.

[69] Chumova J., Kourova H., Trogelova L., Halada P., Binarova P. (2019). Microtubular and Nuclear Functions of gamma-Tubulin: Are They LINCed?. Cells.

[70] Kallai B.M., Kourova H., Chumova J., Papdi C., Trogelova L., Kofronova O., Hozak P., Filimonenko V., Meszaros T., Magyar Z. (2020). gamma-Tubulin interacts with E2F transcription factors to regulate proliferation and endocycling in Arabidopsis. J. Exp. Bot.

[71] Hoog G., Zarrizi R., von Stedingk K., Jonsson K., Alvarado-Kristensson M. (2011). Nuclear localization of gamma-tubulin affects E2F transcriptional activity and S-phase progression. FASEB J..

[72] Binarova P., Dolezel J., Draber P., Heberle-Bors E., Strnad M., Bogre L. (1998). Treatment of Vicia faba root tip cells with specific inhibitors to cyclin-dependent kinases leads to abnormal spindle formation. Plant J..

[73] Kohoutova L., Kourova H., Nagy S.K., Volc J., Halada P., Meszaros T., Meskiene I., Boegre L., Binarova P. (2015). The Arabidopsis mitogen-activated protein kinase 6 is associated with -tubulin on microtubules, phosphorylates EB1c and maintains spindle orientation under nitrosative stress. New Phytol..

[74] Lindstrom L., Alvarado-Kristensson M. (2018). Characterization of gamma-tubulin filaments in mammalian cells. Biochim. Et Biophys. Acta Mol. Cell Res..

[75] Chiba Y., Takei S., Kawamura N., Kawaguchi Y., Sasaki K., Hasegawa-Ishii S., Furukawa A., Hosokawa M., Shimada A. (2012). Immunohistochemical localization of aggresomal proteins in glial cytoplasmic inclusions in multiple system atrophy. Neuropathol. Appl. Neurobiol..

[76] Yam A.Y., Xia Y., Lin H.-T.J., Burlingame A., Gerstein M., Frydman J. (2008). Defining the TRiC/CCT interactome links chaperonin function to stabilization of newly made proteins with complex topologies. Nat. Struct. Mol. Biol..

[77] King M.R., Petry S. (2020). Phase separation of TPX2 enhances and spatially coordinates microtubule nucleation. Nat. Commun..

[78] Petrovska B., Jerabkova H., Kohoutova L., Cenklova V., Pochylova Z., Gelova Z., Kocarova G., Vachova L., Kurejova M., Tomastikova E. (2013). Overexpressed TPX2 causes ectopic formation of microtubular arrays in the nuclei of acentrosomal plant cells. J. Exp. Bot..

[79] So C., Seres K.B., Steyer A.M., Monnich E., Clift D., Pejkovska A., Mobius W., Schuh M. (2019). A liquid-like spindle domain promotes acentrosomal spindle assembly in mammalian oocytes. Science.

[80] Lesca C., Germanier M., Raynaud-Messina B., Pichereaux C., Etievant C., Emond S., Burlet-Schiltz O., Monsarrat B., Wright M., Defais M. (2005). DNA damage induce gamma-tubulin-RAD51 nuclear complexes in mammalian cells. Oncogene.

[81] Oshidari R., Strecker J., Chung D.K.C., Abraham K.J., Chan J.N.Y., Damaren C.J., Mekhail K. (2018). Nuclear microtubule filaments mediate non-linear directional motion of chromatin and promote DNA repair. Nat. Commun..

[82] Pilhofer M., Ladinsky M.S., McDowall A.W., Petroni G., Jensen G.J. (2011). Microtubules in Bacteria: Ancient Tubulins Build a Five-Protofilament Homolog of the Eukaryotic Cytoskeleton. PLoS Biol..

